# Association between *TNF-alpha* polymorphism and the age of first suicide attempt in chronic patients with schizophrenia

**DOI:** 10.18632/aging.102692

**Published:** 2020-01-18

**Authors:** Xiaoe Lang, Tammy H. Trihn, Hanjing Emily Wu, Yongsheng Tong, Meihong Xiu, Xiang Yang Zhang

**Affiliations:** 1 Department of Psychiatry, The First Clinical Medical College, Shanxi Medical University, Taiyuan, Shanxi Province, China; 2 Department of Psychiatry and Behavioral Sciences, The University of Texas Health Science Center at Houston, Houston, TX 77030, USA; 3 Peking University HuiLongGuan Clinical Medical School, Beijing HuiLongGuan Hospital, Beijing, China; 4 CAS Key Laboratory of Mental Health, Institute of Psychology, Chinese Academy of Sciences, Beijing, China; 5 Department of Psychology, University of Chinese Academy of Sciences, Beijing, China

**Keywords:** schizophrenia, genotype, suicide, TNF-alpha, association

## Abstract

Patients with schizophrenia (SCZ) exhibit higher suicide rates than the general population. However, the molecular mechanism remains poorly understood. *Tumor necrosis factor (TNF)-alpha* polymorphisms have been repeatedly indicated to play a pathogenetic role in various mental disorders, but none of these studies focused on the susceptibility to suicidal behavior in SCZ. We recruited 1087 chronic inpatients with SCZ and 576 controls to assess the psychopathological symptoms of SCZ using the Positive and Negative Syndrome Scale scales. We selected 2 polymorphisms (-308G>A and -1031C>T) in the *TNF-alpha* gene and analyzed their associations with SCZ and suicide. Our results showed that *TNF-alpha* -308G>A and -1031C>T were not related to SCZ and suicide. However, we found that suicide attempters with the C allele carriers exhibited suicidal behaviors significantly later than those with TT genotype in the SCZ patients. The haplotype containing the T allele of the -1031 was significantly associated with the age of suicide initiation. Further logistic regression analysis showed that -1031C>T interacted with psychopathological symptoms and drinking, age of smoking, and related to the initiation age of suicide attempts. Our study demonstrated that the *TNF-alpha* variants may affect the age at which suicide attempts started among SCZ suicide attempters.

## INTRODUCTION

Patients with schizophrenia (SCZ) have a high risk of attempting and completing suicide. Suicide is considered as one of the most common causes of premature death in SCZ patients [1]. The average life expectancy of patients with SCZ is estimated to have decreased by approximately 14.6 years [2]. However, the pathophysiological mechanisms for the high rate of suicidality in SCZ are still unclear.

Recent findings have recognized the possible role of the abnormal immune system in the primary pathophysiological mechanism of suicidal behavior [3–4]. It is hypothesized that cytokines might produce behavioral alterations through the impact on neuronal integrity, synaptic remodeling, neurogenesis and neurocircuity [5–6]. Emerging evidence demonstrated that abnormal expression of pro-inflammatory cytokines may lay a critical role in the etiology and pathogenesis of suicide. For example, previous studies found higher levels of the soluble interleukin-2 receptor (IL-2R) in the plasma of suicide attempters [7] and increased IL-6 in cerebrospinal fluid (CSF) in persons who attempted suicide than controls [8]. The studies in postmortem brain samples from suicide victims showed elevated levels of IL-13 and IL-4 [9] in the orbitofrontal cortical area and tumor necrosis factor (TNF)-alpha in the anterior prefrontal cortex [10]. Regarding the associations between inflammation and suicidal behavior, several recently published meta-analyses summarized the new data and provided more information on inflammatory changes in suicidal attempters [11–12].

Several lines of studies of peripheral biomarkers have shown higher serum TNF-alpha levels in those persons with suicide attempts, suicidal ideation, or suicide [10–13], but lower levels in a report of depressed persons with high suicidal ideation (including recent suicide attempters), in contrast to depressed persons without suicide ideation [14]. These results of alteration in serum TNF-alpha levels were also supported by accumulating studies in the dorsolateral prefrontal cortex of the postmortem brains. The studies showed that TNF-alpha expression was significantly higher in subjects who committed suicide regardless of the type of psychosis diagnosed, comparing to those who died due to causes other than suicide [10, 15]. Although many studies support TNF-alpha in suicide, there are a handful of studies that showed no group differences in blood levels or expression of TNF-alpha [16–18]. In particular, a recent meta-analysis revealed that levels of IL-1β and IL-6 were most robustly associated with suicidality, but no association was found between TNF-alpha levels and suicidal behaviors [19]. Although several pieces of literature had also examined the association between the polymorphism of other cytokine gene and suicidal behavior in major depression disorder (MDD) [20–21], there is no current research that has analyzed the relationship between *TNF-alpha* polymorphism and suicide in SCZ. At this time, only the -308G>A polymorphism in the *TNF-alpha* gene has been studied in relation to suicide attempts in individuals with MDD [21].

Inflammation is not only related to suicidal behavior, but also to mental disorders. Many studies by our team and others have suggested that the pathogenesis of schizophrenia involves an alteration of peripheral immune system that leads to altered blood levels of TNF-alpha [22–23]. A recent meta-analysis found alteration of TNF-alpha serum levels persist following treatment with clozapine [24]. *TNF-alpha* gene has multiple frequent polymorphisms [25] and several common polymorphisms were related to SCZ [26–27]. Our previous studies in chronic patients with SCZ found that C allele carriers of rs1799964 polymorphism in the *TNF-alpha* gene displayed better cognition functions [28] and decreased TNF-alpha serum levels were significantly associated with positive symptoms and general psychopathology subscores [29]. Taken together, all these studies demonstrate that TNF-alpha plays critical roles in the pathophysiological mechanism of SCZ.

Considering the high suicide rate in the patients with SCZ and the pathogenic role of *TNF-alpha* gene and alteration of TNF-alpha serum levels in SCZ, it would be beneficial to investigate the association between the *TNF-alpha* gene and suicidality of SCZ patients. Based on previous literature, we selected -308G/A (rs1800629) and -1031T/C (rs1799964) single nucleotide polymorphisms (SNPs) to assay among the patients with SCZ in this study. -308G/A polymorphism has been confirmed by several studies to be a susceptibility factor for suicide in patients with MDD [21], while -1031T/C polymorphism was confirmed by our previous study to be related to cognitive impairments in the patients with SCZ [28]. Thus, we hypothesized that the 2 tag SNPs of TNF-alpha gene might confer the risk for suicidal attempt in SCZ, and then examined whether the interplay of gene and environment might affect the risk of suicidal behavior in SCZ patients.

## RESULTS

### Demographic and clinical characteristics subgrouped by attempted suicide

The demographic characteristics and clinical data are shown in Table 1. The participants were unequally distributed in terms of sex, age, BMI, and smoking status between SCZ patients and controls (all p < 0.01; Bonferroni corrected all p>0.05). In the subgroup analysis based on attempted suicide, significant differences were found in age, the number of the cigarettes smoked each day, the age of smoking onset, and the general psychopathology subscores of PANSS (all p< 0.05; Bonferroni corrected all p>0.05). These significantly different variables in the subgroup analysis were adjusted in the subsequent analyses. A few of the subjects did not have the clinical measures or the questionnaire fully, so numbers may vary slightly in different categories.

**Table 1:** Demographic characteristics, clinical data in suicide attempters and non-attempters of schizophrenia and healthy controls.

**Variable**	**HC (n=576)**	**SZ (n=1087)**	**F or *χ^2^* (p)**	**Suicide (n=152)**	**Non-Suicide (n=805)**	**F or*χ^2^* (p)**
Gender (male/female)	263/313	890/197	232.3 (<.01)	128/24	655/150	0.69(.40)
Age (years)	45.8±12.8	47.8±10.2	11.7 (<.01)	44.8±10.6	48.4±10.1	3.8(<.01)
Education (years)	8.5±3.2	9.3±6.4	2.1 (.05)	8.9±2.9	9.3±6.9	0.75(.45)
Smokers (%)	37.9%	66.3%	51.3(<.01)	71.1%	65.0%	2.1(.086)
BMI (kg/m^2^)	25.1±3.9	24.5±3.9	2.7 (<.01)	24.4±3.9	24.6±4.0	0.46(.65)
NSC	14.7±11.2	3.8±2.8	89.7(<.001)	4.6±3.7	3.7±2.7	2.12(.02)
Age of smoking onset	24.1±9.5	21.8±3.9	5.6(<.001)	19.2±3.1	21.9±4.0	2.51(.015)
Age of onset		23.5±5.4		23.7±6.5	23.5±5.5	0.31(0.76)
PANSS						
Positive subscore		11.7±4.9		12.6±4.9	11.8±5.2	1.4(.24)
Negative subscore		22.9±8.2		20.6±7.1	22.3±8.4	2.7(.10)
General psychopathology subscore		26.4±6.5		27.0±6.1	25.4±5.8	4.4(.036)
Total score		61.0±14.9		61.4±14.4	59.8±15.2	.17 (.68)
TNF-a genotype frequency						
-308 (AG/GG)	66/498	105/762	.05(.82)	15/106	75/558	.03(.88)
A	66	105	.02(.93)	15	75	.03(.88)
G	1062	1649		227	1191	
-1031 (CC+CT/TT)	230/341	375/567	.86(.45)	45/82	247/438	.02(.89)
C	230	375	.03(.89)	45	247	.01(.90)
T	912	1509		209	1123	

Note: SZ: schizophrenia, HC: healthy control, BMI: body mass index, PANSS: positive and negative syndrome scale, NSC: number of smoking cigarette.

### Associations of TNF-alpha gene polymorphisms with schizophrenia and suicide

No deviation from Hardy-Weinberg equilibrium was found in the patients and controls, or in the subgroup analysis based on suicide attempt. No significant differences in the genotype and allele frequencies between patients and controls were found for *TNF-alpha* -308G>A (rs1800629) and -1031C>T (rs1799964). Also, there was no genotypic or allelic correlation between the two SNPs and attempted suicide (Table 1). In a stepwise logistic regression analysis for relationships between attempted suicide and *TNF-alpha* polymorphisms, with age, gender, education, BMI, smoking, and clinical symptoms of schizophrenia as covariates, it was of particular interest to show that -1031C>T genotype distributions (beta = 0.65, df = 1, Wald χ^2^ = 4.3, p = 0.024) and PANSS total scores (beta = 0.032, df = 1, Wald χ^2^ = 8.9, p = 0.003) reached significantly different values in the subgroup analysis. However, the association between -308G>A and suicide still was not statistically significant after a logistic regression analysis (p>0.05). Given that most of the samples were male patients, we performed stratified analysis for each sex and still did not find significant differences in the allele and genotypic frequencies of the two SNPs between male suicide attempters and male non-attempters or female attempters and female non-attempters (all p>0.05).

### Associations between the TNF-alpha polymorphism genotype and haplotype and clinical variables in the patients with attempted suicide

To investigate the correlation between *TNF-alpha* polymorphism and clinical variables, the quantitative trait test was used to determine the relationship between *TNF-alpha* -308G>A or -1031C>T and individual phenotype. We found no association between -308G>A or -1031C>T and any clinical phenotypes, including gender, age, education, onset of illness, BMI, antipsychotic treatment (type, dose and duration of treatment), hospitalization, duration of illness as well as the symptoms of schizophrenia that were measured by the PANSS (all p>0.05; Table 2)

**Table 2:** Demographic and clinical characteristics of suicide attempters based on the TNF-α -308A/G and −1031T/C genotype.

	**-1031 (T/C)**		**t or**	**p-**	**-308 (A/G)**		**t or**	**p-**
	**TT**	**TC+CC**	**χ2**	**Value**	**GG**	**AG**	**χ2**	**Value**
Male/female	72/10	38/7	.60	.39	94/12	13/2	.69	.55
Age(years)	47.1±9.2	45.9±8.9	.24	.81	48.1±9.6	46.8±8.9	1.32	.19
BMI (kg/m2)	24.5±3.5	25.2±4.9	.82	.42	24.4±4.1	25.8±2.9	1.04	.30
Education(years)	8.5±2.8	8.7±1.8	.67	.20	8.6±2.7	8.3±2.0	.42	.68
Onset age(year)	23.7±6.5	24.1±6.4	.05	.82	24.0±6.0	21.9±3.0	1.31	.19
Hospitalization	4.4±2.8	4.4±2.4	.00	.98	4.3±2.4	4.5±1.7	.09	.76
Duration (years)	24.9±9.3	24.2±9.7	1.02	.31	24.6±9.4	23.7±9.2	.80	.42
Medication (mg)	475.3±464.9	436.5±299.4	1.26	.21	469.6±390.6	385.7±191.9	1.36	.18
PANSS scores								
P subscore	13.7±5.1	13.0±5.2	.53	.60	13.2±5.3	12.8±4.3	.16	.87
N subscore	22.6±7.4	22.6±6.3	.04	.97	23.7±6.9	21.3±3.1	.81	.42
G subscore	28.6±5.7	27.7±6.7	.54	.59	28.0±6.5	30.5±5.5	.88	.38
Total score	65.3±13.6	63.0±15.1	.59	.56	65.0±15.1	64.7±11.5	.05	.96
**TFS (years)**	**29.2±8.2**	**34.0±9.7**	**5.2**	.025*	31.7±9.1	32.6±9.8	.327	.75
NSA	1.4±0.9	1.6±0.9	.57	.57	1.5±0.8	1.7±1.2	.93	.36

Note: Medication (mg) means “mean antipsychotic dose (in chlorpromazine equivalents)”; BMI, body mass index; TFS, time of first suicide; NSA, number of suicide attempts. *indicate a significant difference in age of onset.

### The related factors influencing the initiation age of attempted suicide

**Figure 1:**
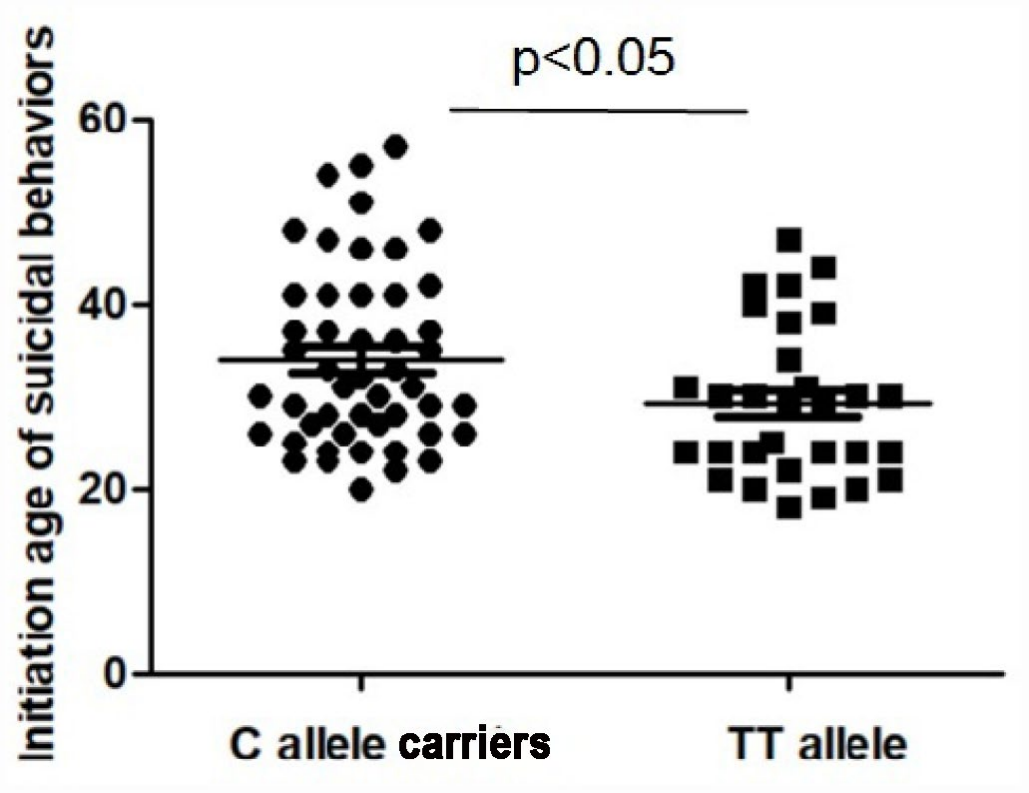
**The initiation age of suicidal behaviors in the patients with schizophrenia.** The sample means are indicated by the black bars. The initiation age of suicidal behaviors was significantly higher in C allele carriers than those with TT allele (p < 0.05).

As shown in Table 2 and Figure 1, the initiation age of attempted suicide was significantly different in TT group and C allele carries group of -1031C>T (29.2 ± 8.2 years vs 34.0 ± 9.7 years, F=5.2, p = 0.025; Bonferroni corrected p>0.05). Among suicide attempters, subjects with the C allele demonstrated initial suicidal behaviors significantly later than those with the TT genotype (p<0.05; Bonferroni corrected p>0.05). However, the onset age of first suicide were not different based on -308G>A genotype grouping. Multiple stepwise regression analysis, including -1031C>T genotype, age, onset age, duration of illness, the number of hospitalizations, antipsychotic drugs (dose equivalent to chlorpromazine), PANSS and its subscale scores, and age at attempted suicidal initiation (as dependent variable) in suicide attempters of SCZ patients identified PANSS general psychopathology subscore (beta = -0.069, Wald *χ*^2^ = 9.93, p = 0.002) and -1031C>T genotype (beta = 0.66, Wald *χ*^2^ = 5.02, p = 0.025) to be related to age at suicide attempt initiation.

## DISCUSSION

This is the first investigation to explore the relationship between *TNF-alpha* gene and risk for attempted suicide in Han Chinese patients with SCZ, showing no relationship between *TNF-alpha* gene -308G>A and -1031C>T polymorphism and SCZ and suicide attempt. However, we found a link between the -1031C>T polymorphism and the age at which SCZ patients began to attempt suicide. Moreover, the age of suicide initiation was significantly positively correlated with age of symptoms onset in the patients.

Several studies have investigated the association of polymorphisms in the *TNF-alpha* gene and SCZ, but with inconsistent results [27, 30–33]. In the present study, both -308G>A and -1031C>T polymorphisms were not associated with SCZ, consistent with some of these studies [34–36], especially a study from China [37]. Moreover, we did not reveal a relationship between the 308G-1031C haplotype and SCZ. Inconsistent with our findings, a recent study in Poland found significant associations between the haplotype of *TNF-alpha* gene 1031T-863A-857C-308G, 1031C-863C-857C-308G, and 1031C-863C-857T-308G and increased risk of SCZ [33]. A possible mechanism for these inconsistent results is that the patients recruited in the study by Suchanek-Raife et al. [33] were different than those in our study (paranoid subtype of SCZ vs SCZ). Another explanation for the difference between *TNF-alpha* polymorphism and SCZ might be due to ethnic differences, as the frequency of -1301C>T and -308G>A genotypes varied significantly between different ethnicities.

Regarding the relationship of suicide attempt and the *TNF-alpha* gene, our results indicate that there was no association between two tag SNPs in the *TNF-alpha* gene and suicide attempt. The patients with SCZ were associated with higher risk of suicide than the general population, and previous studies speculated that the pathophysiology of suicide might originate as a disturbance within the immune system [38–39]. However, only a few groups have studied the potential role of *TNF-alpha* gene in suicide. Results from these groups showed that -308G>A was related to suicidal behaviors in MDD [20–21] and the -308G was correlated with the number of suicide attempts in the patients with psychiatric diseases. Up until now, only one previous study has attempted to explore the relationship between *TNF-alpha* gene polymorphism and suicide in SCZ [33]. However, neither the above study nor our study reported a significant association between suicide and *TNF-alpha* gene polymorphism. Furthermore, neither study showed any association between suicide and *TNF-alpha* haplotype in patients. We attributed the failure to find a relationship between suicide and *TNF-alpha* polymorphism to low sample size of suicidal patients with SCZ in both studies, since there were only 120 suicide attempters in this study and 74 attempters in the above study for the genetics analysis. The sample size of suicide attempters was small, resulting in an even smaller number of patients found to have the CC genotype of -1031 polymorphism, which may produce false negative results. Therefore, additional studies with larger samples containing several ethnic populations are needed to confirm these findings. It is worthy of mentioning that in a stepwise logistic regression analysis for relationships between attempted suicide and *TNF-alpha* polymorphisms, with age, gender, education, BMI, smoking and clinical symptoms shown on PANSS scores as covariates, we found that -1031C>T genotype distributions (beta = 0.65, df = 1, Wald χ^2^ = 4.3, p = 0.024) and PANSS total score (beta = 0.032, df = 1, Wald χ^2^ = 8.9, p = 0.003) reached significance. This result suggests that under the influence of clinical variables, combined with clinical psychopathological symptoms, the *TNF-alpha* -1031C>T polymorphism may play a role in attempted suicide in SCZ patients. Why was there no significant difference in the allele or genotype distribution of *TNF-alpha -*1031C>T polymorphism between suicide attempters and non-attempters in SCZ patients, and then this difference became significant when combined with clinical variable, especially psychopathological symptoms? However, due to our cross-sectional design in this study, we are currently unable to provide a reasonable explanation for this phenomenon, which deserves further exploration in future research.

Another important finding was that the -1031C/T polymorphism may have an effect on the age at which suicidal behavior initiation occurred in SCZ. Patients attempting suicide with the -1031C allele started committing suicide later than those who carried the TT genotype. Further haplotype analyses found that the age at which suicidal behavior initiated in patients carrying 308(A)-1031(T) was earlier than those with haplotype counterparts, which further supports that the -1031TT genotype may be associated with earlier age of suicide initiation. Suicide is a complex behavior, and recent scientific literatures analyzed the age of suicide initiation of suicidal attempters as a potential candidate marker to characterize different subgroup attempters and reduce the heterogeneity of suicidal behavior [40–42]. These studies suggested that the age of the first suicide attempt might represent an effective candidate symptom for the future study of genetic susceptibility in suicidality [41].

It would be extremely meaningful to explore the possible mechanism underlying the associations between the *TNF-alpha* -1031 TT genotype and the earlier age at which suicide initiation occurred in the patients with SCZ. Many studies have indicated that the two tag SNPs (-1031C>T and -308G>A polymorphisms) have an effect on the protein production; although these results were contradictory [43]. For example, one study demonstrated that the T allele of the -1031C>T polymorphism led to lower plasma levels in diabetic patients [44]. Another study in chronic HBV patients found that patients with -308 GG genotype had lower plasma levels of TNF-alpha, as compared to those with other genotypes [45]. Moreover, the results from the haplotype including 2 tag SNPs also have indicated to influence the levels of TNF-alpha in preterm infants [46]. Taken together, all these studies suggested that -1031 TT genotype was related to lower levels of TNF-alpha in the plasma. We speculated that the association may be related to the direct effects of the -1031C>T and -308G>A polymorphisms themselves on the levels of TNF-alpha in the immune system and brain. Lower levels of TNF-alpha in the patients may suggest an induction defect of the inflammatory pathway or inhibition of the activity of the cytokine. Abnormal levels of TNF-alpha and other cytokines at critical times in the development of neural system and functions, such as cell signaling transduction, neurogenesis, neurotransmission, synaptic plasticity, and behaviors, may make individuals susceptible to SCZ or other related diseases [47–51]. However, no studies have shown the relationship between aberrant TNF-alpha and suicidality in SCZ, but the results from the patients with depression could provide additional evidence. A previously published literature on acutely suicidal depressed adolescents (including the recent suicide attempters) showed a significant reduction in TNF-alpha levels as compared to depressed non-suicidal subjects [14]. In particular, another study showed that *TNF-alpha* mRNA was associated with impulsivity and hopelessness after controlling for demographics and substances used [52]. In addition, the study from our group showed a decrease in serum levels of TNF-alpha in patients with SCZ compared with controls, so it is reasonable to conclude that TNF-alpha may be involved in suicidal behaviors in SCZ [29]. Taken together, all of the results above suggested that the -1031TT genotype was related to lower levels of TNF-alpha in plasma and might be involved in the pathogenesis of suicidal behaviors in patients with SCZ.

On the other hand, our previous results demonstrated that -1031C/T polymorphism was related to the onset age of SCZ in long-term hospitalization patients with SCZ [28]. Interestingly, our analysis found that the age of suicide initiation was positively correlated with the age at which psychopathological symptoms appeared in the patients with SCZ. This indicated that the patients with the TT genotype had reduced TNF-alpha levels and an earlier onset age of schizophrenia and suicide initiation. The underlying mechanisms of this positive correlation are unclear, but suicidal behavior appeared after the onset of symptoms in chronic patients with SCZ, implying that suicidal behavior might be an aspect of schizophrenia as a psychiatric illness or be a consequence of depression symptoms seen in these patients. Subjects with the TT genotype initiated suicide earlier, suggesting that the decreased TNF-alpha levels related to the TT genotype affected pathological symptoms earlier and more severely. Studies in animal models indicated that *TNF-alpha* gene knockout mice showed increased emotional responses when exposed to stress compared with wild-type mice [53]. Hence, it is possible that the underlying mechanism of the association between lower levels of TNF-alpha mediated by the T allele of the -1031T/C polymorphism and earlier age of suicide attempt initiation might lie in the neurotransmitter pathways related to suicide in patients with chronic SCZ. Indeed, many studies showed that the reduction of TNF-alpha could significantly affect the levels of monoaminergic neurotransmitters, such as serotonin, dopamine, and glutamine [54–56]. Animal studies showed that systemic administration of TNF-alpha caused an increase in serotonin (5-HT) levels in the prefrontal cortex (PFC) [57], while postmortem studies found PFC serotonergic system abnormalities in suicide completers [15, 58]. Furthermore, reduced TNF-alpha levels caused aberrantly regulated cytokines, and activation of the key enzyme guanamine-2,3-dioxygenase (IDO), which subsequently catalyzed the production of kynurenine (KYN) from tryptophan (TRP) [13]. Activation of the TRP/KYN metabolic pathway reduced the production of serotonin, affecting serotoninergic and glutamatergic neurotransmission which play a role in mood and suicide risk. Both KYN and TRP can enter the brain through the blood-brain barrier (BBB) (Schwarcz et al., 2012), where they are catabolized by either microglia or astrocytes into a variety of neuroactive compounds [59]. A recent study by Bradley et al. revealed a 40% reduction in plasma TRP levels and a 40% elevation in the KYN/TRP ratio in MDD patients attempting suicide, compared to non-attempt patients with MDD and control subjects (Bradley et al., 2015). However, the exact mechanism linking the TNF-alpha gene and age at suicide initiation is unclear and deserves further investigation.

The study has several limitations that should be noted. First, although the sample was large, only 152 patients with suicidal behaviors were included. Therefore, the relationship between *TNF-alpha* gene and suicidal tendencies in SCZ was preliminary, and further validation needs to be carried out in a larger, independent sample to increase statistical capacity before a definitive conclusion can be drawn. Second, there are several known polymorphisms in the promoter of *TNF-alpha* gene, but we had measured only two of those polymorphisms. Whether the other variants affected the results of this study is unclear. A complex analysis of the influences of polymorphisms in the gene should be examined in further researches. Third, the levels of TNF-alpha and other inflammatory cytokines in the cerebrospinal fluid and blood were not measured in this study. Therefore, we were unable to assess whether the T allele of the -1031T/C caused a decrease in TNF-alpha levels in the brain. Fourth, one of the major limitations of the current study is short of data on suicide method and associated lethality. Therefore, suicide attempts were not classified as serious suicide attempts. Therefore, we were able to only explore various factors related to suicide attempts, rather than the severity of the suicidal behaviors. In further investigations, an appropriate tool to evaluate the severity of suicide attempts would be warranted. Fifth, the hidden demographic stratification in our sample may be a confounding factor. We had a small number of female patients that may cause a discrepancy in results. Sixth, all SCZ patients enrolled in this current study were inpatients with long-term duration of illness. Hence, our findings were limited to chronic inpatients with longer duration of symptoms and more severe psychopathological symptoms compared to the typical psychiatric outpatients. Therefore, our findings in this study could not be generalized to other patients. Seventh, it is important to tease out how the genetic variation at the specific locus is getting translated into a phenotypic variation. However, we did not test the functional relevance of -1031C/T by using an in vitro cellular model, which should be remedied in the future study. Finally, although previous study showed that the C allele of TNF alpha-1031C/T was associated with higher expression of TNF-alpha (El-Tahan et al 2016), we did not measure TNF-alpha levels in this study. It would be better if the blood level of TNF-alpha could be provided in the same patient sample to increase the validity of this paper. Thus, we could not provide the mechanistic explanation linking the C allele to later age of suicide attempt in SCZ patients, which deserves further investigation.

In conclusion, we found that the -1031C>T and -308G>A in the *TNF-alpha* gene did not appear to modify the propensity to develop schizophrenia and suicidal behaviors, but they did influence the age of suicide initiation in patients with SCZ. In addition, environment factors, such as drinking status, smoking, and psychotic symptoms, interacted with *TNF-alpha* gene and appeared to be related to the age at suicide initiation in SCZ. This suggests that the interaction between the genetics and environment might influence some pathological aspects of suicide in SCZ. However, our results may have appeared by chance, due to limited sample size of suicide subgroup. Therefore, the current association finding in this study should be confirmed in other independent samples.

## MATERIALS AND METHODS

### Subjects

1087 inpatient with schizophrenia were randomly recruited from Beijing HuiLongGuan hospital and HeBei Province Veteran Psychiatric Hospital. The current study was conducted from August 2015 to September 2017. All patients were of the chronic type, with duration of illness for at least 5 years. The inclusion criteria in the study were as our previous studies in detailed descriptions: age 20-75 years; and diagnosis of SCZ according to the Structured Clinical Interview for DSM-5 (SCID) by the trained psychiatrists. All subjects had been taking stable doses of oral antipsychotic medication for at least one year prior to recruitment. Antipsychotic medications consisted mainly of drug monotherapy, including clozapine (n = 488), risperidone (n = 239), chlorpromazine (n = 79), sulpiride (n = 55), perphenazine (n = 51), quetiapine (n = 47), haloperidol (n =38), aripirazole (n = 32), and others (n = 58). According to the method provided by Woods [60], the average antipsychotic dose (in terms of chlorpromazine equivalent) was 438 ± 407 mg / day.

According to the definition of the World Health Organization (WHO 2014), the above four psychiatrists evaluated the history of suicide attempts based on chart review and interview data. Attempted suicide refers to any non-fatal suicide, which is intentional self-inflicted poisoning, injury, or self-harm, with or without fatal intentions or consequences (WHO 2014). In this study, there were 152 SCZ patients with a history of suicidal attempt and suicidal ideation and 935 SCZ patients without suicidal behaviors. The mean number of attempted suicide was 1.51 ± 0.94 times (ranging from 1 time to 5 times).

In this study, 576 healthy controls were recruited. They were randomly recruited through advertisements in the local community, and they matched patients with fewer years of education. Patients and healthy controls had comparable socioeconomic backgrounds. Psychiatrists used the standardized SCID diagnostic assessment to rule out controls with Axis I disorders. Individuals taking psychoactive drugs (e.g. antipsychotic, anti-anxiety, antidepressant or mood stabilizing drugs) were also excluded.

The current study was approved by institutional Review Board of Beijing HuiLongGuan hospital. All subjects provided signed informed consent form to participate in this study.

### Clinical measures

On the same day of blood drawing, the symptoms of schizophrenia were assessed by PANSS. Four psychiatrists participated in a training session on the use of PANSS and conducted repeated assessment tests prior to the study. In this present study, a five-factor model of SCZ symptoms was analyzed, labeled as ‘positive factor’, ‘negative factor’, ‘cognitive factor’, ‘depression factor’, and ‘excitement factor’ [61].

### TNF-alpha polymorphisms analysis

Based on previous published studies, we selected 2 tag SNPs in the *TNF-alpha* gene based on previous studies, -308G>A (rs1800629) and -1031C>T (rs1799964). These two polymorphisms were genotyped as the protocol described in our previous study [62]. The primers and extent probe of -1031C>T were designed as follows (sense: 5′-TATGTGATGGACTCACCAGGT-3′, antisense: 5′-CCTCTACATGGCCCTGTCTT-3′, probe: CAGAGCGCTAAACCC). The primers and extent probe of -308G>A were sense: 5′-TGTGACCACAGCAATGGGTAGGAGA-3′, antisense: 5′-CCCAGTGTGTGGCCATATCTTCTTA-3′, probe: TCGAGTATGGGGACCCCC.

A technician who was blind to clinical conditions genotyped each DNA sample of all subjects twice for the accuracy of genotyping. For quality control, 5% of all samples were randomly selected for repeated genotyping. Quality control tests showed that the error rate of regenotyping data was less than 0.1%.

### Statistical analysis

Adherence to Hardy-Weinberg (HW) equilibrium was evaluated by SHEsis (http://analysis.bio-x.cn). Analysis of variance (ANOVA) for continuous variables and the Chi-Square test (χ^2^ test) for categorical variables were used to analyze the differences in patients and controls. ANOVA and *χ^2^* test were used to find the associations between demographic and clinical variables and the suicidal behaviors. The differences in allele and genotypic frequencies of -308G>A and -1031C>T polymorphisms between patients and healthy controls and between suicide attempters and non-attempters within the patient group were tested by the *χ^2^* test. SHEsis software was utilized to analyze pairwise LD statistics for two tag SNPs, haplotype frequency, haplotype block, and haplotype association with schizophrenia or suicidal behavior. Quantitative trait tests were conducted to analyze the relationship between gene polymorphisms and characteristics of suicidal behavior. Less than 1% of rare haplotypes were excluded in the association analysis. The effects of the *TNF-alpha* genotypes on the demographic characteristics and clinical symptoms in schizophrenia attempters were examined by ANOVA and effects of genotypes on the age of suicide initiation were examined by one-way analysis of covariance (ANCOVA) to adjust for clinical confounding factors using the SPSS 18.0 software. Bonferroni corrections were used to each test to adjust for multiple testing.

Furthermore, we used binary logistic regressions to calculate *TNF-alpha* genotypic association with suicidal behavior within the patient group while considering recessive, dominant and codominant genetic models. Only the significant genetic models in the binary logistic regression and those variables with significant associations in the ANOVA analyses between schizophrenia attempters and non-attempters were included in the stepwise multiple logistic regression analyses. In the model, the genetic models and confounders were used as independent variables, and the diagnosis was used as a dependent variable.

Statistical power of the sample was computed using Quanto Software with known risk allele frequencies and the suicide attempt prevalence in the patients with schizophrenia.
